# *CCR5*Δ32 polymorphism is associated with increased central memory CD4+ T cells in virologically suppressed people living with HIV on antiretroviral therapy

**DOI:** 10.1007/s00430-026-00872-4

**Published:** 2026-04-06

**Authors:** Henrique Fernando Lopes-Araujo, Wlisses Henrique Veloso Carvalho-Silva, José Leandro Andrade-Santos, Maria Carolina Santos Guedes, Fabrício Oliveira Souto, Luiz Cláudio Arraes De Alencar, Isadora Bandeira De Luna Paes Barreto Brennand, Thayana Karinne Oliveira Monteiro, Kleyverson Feliciano-Santos, José Artur Bogo Chies, Rafael Lima Guimarães

**Affiliations:** 1 Department of Genetics, Federal University of Pernambuco - UFPE, Recife, Pernambuco, 50670-901 Brazil; 2 Keizo Asami Institute (iLIKA), Federal University of Pernambuco - UFPE, Recife, Pernambuco, 50670-901 Brazil; 3 Aggeu Magalhães Institute (IAM) - Oswaldo Cruz Foundation (FIOCRUZ), Recife, Pernambuco, 50740-465 Brazil; 4 Agreste Academic Center, Federal University of Pernambuco - UFPE, Caruaru, Pernambuco, 55014-900 Brazil; 5 Institute of Integral Medicine Professor Fernando Figueira (IMIP), Recife, Pernambuco, 50070-550 Brazil; 6 Department of Genetics, Federal University of Rio Grande do Sul - UFRGS, Porto Alegre, 91501-970 Rio Grande do Sul Brazil

**Keywords:** ART, *CCR5*delta32, Genetic polymorphism, CD4 + T cell subsets, Immune reconstitution

## Abstract

The *CCR5Δ32* polymorphism is a 32-base pair deletion in the *CCR5* gene that has been associated with slower HIV disease progression. However, its role in immune reconstitution during antiretroviral therapy (ART) remains unclear. We analyzed 236 virologically suppressed people living with HIV (PLHIV) after 24 months of ART, comprising 217 individuals homozygous for the wild-type *CCR5* allele and 19 heterozygous carriers of *CCR5Δ32*. Heterozygotes exhibited a significantly higher frequency of central memory CD4+ T cells compared with wild-type homozygotes (40.33 ± 8.79 vs. 32.71 ± 7.06; *p* = 0.0196), along with a tendency toward increased effector CD4+ T cells (5.100 [1.818–7.588] vs. 2.260 [1.485–3.503]; *p* = 0.0459). In contrast, longitudinal follow-up revealed that wild-type homozygotes achieved higher absolute CD4+ T cell counts at both 18 and 24 months of ART (*p* < 0.05). These results suggest that *CCR5Δ32* contributes to qualitative preservation of CD4+ T cell subsets while limiting quantitative immune reconstitution, thereby providing novel insights into the long-term immunological impact of this polymorphism in virologically suppressed PLHIV.

## Introduction

The human immunodeficiency virus (HIV) is a lentivirus within the Retroviridae family [1, 2]. HIV primarily infects and ultimately lead to the destruction of cells of the immune system, mainly CD4+ T cells, which results in an immunocompromised environment [3]. The final stage of HIV infection is acquired immunodeficiency syndrome (AIDS), where people living with HIV (PLHIV) are committed by high uncontrolled viremia levels and severe immunodepression (< 200 CD4+ T cells/µL) allowing the development of opportunistic infections and malignancies [4, 5]. Antiretroviral therapy (ART) is the major strategy to suppress viral replication to undetectable levels (< 50 copies of viral RNA/mL), enabling immune reconstruction. However, CD4+ T cell recovery varies considerably among individuals, and host genetic factors may influence the reconstitution of these cells in PLHIV on ART [6, 7].

HIV cellular infection dynamics is regulated by a series of molecular interactions between the virus and host cells. The viral envelope glycoprotein gp120 binds to the CD4 receptor and subsequently interacts with either the CCR5 or CXCR4 coreceptor for the HIV entry [8, 9]. This interaction induces conformational modifications in another viral glycoprotein, gp41, promoting the fusion of the viral envelope to the cellular membrane [10]. CCR5 is highly expressed on memory CD4 + T cells and CXCR4 is expressed on both memory and naive CD4+ T cells. Thus, HIV primarily targets CD4+ T cells, but it can also infect other immune cells, for example: macrophages and dendritic cells that also express these receptors [11].

As mentioned, CCR5, also known as C-C chemokine receptor 5, is expressed in different immune cells, including lymphocytes, granulocytes, and monocytes [12]. This receptor is important for immune surveillance and inflammatory response by regulating the migration and functional activity of memory and effector T cells, macrophages, and dendritic cells [13, 14]. Several chemokines bind to CCR5, including CCL3, CCL4, and CCL5, which can block HIV entry to host cells and prevent infection. Beyond its role in HIV infection, CCR5 also modulates immune response in a variety of inflammatory conditions, including other viral infections, autoimmune diseases, and cancer [15].

The *CCR5* gene, located on chromosome 3 (3p21.31), encodes the CCR5 protein and consists of two introns and three exons involved in the production of this chemokine receptor. There is a 32-base pair deletion in exon 3, known as *CCR5*Δ32 (rs333) allele, that is relatively frequent in certain populations worldwide, particularly those from north Europe and of European ancestry [16, 17]. This polymorphism occurs in the region encoding the second extracellular loop, leading to a premature stop codon and the production of a truncated protein that prevents CCR5 expression on the cell surface [18, 19]. Homozygous individuals for the deletion (Δ32/Δ32) usually lack expression of CCR5, and heterozygotes (wt/Δ32) exhibit reduced expression of this receptor [15]. In Brazil, *CCR5*Δ32 allele frequency is found in averages from 4% to 6%, varying by region due to the country’s diverse ancestry and admixed population [20, 21]. By reducing CCR5 expression, *CCR5*Δ32 polymorphism acts as a resistance factor against HIV entry, and slows disease progression. It has also been associated with a lower risk of HIV-related lymphoma. However, diminished CCR5 expression on immune cells may impair chemokine signaling and potentially alter immune responses [22, 23].

Although ART controls viremia, immune reconstitution remains variable among PLHIV undergoing ART [24]. Therefore, this study investigated the influence of *CCR5*Δ32 on the frequency of different CD4+ T cell subsets in virologically suppressed PLHIV receiving ART, aiming for a better understanding of the immune response in these individuals.

## Materials and methods

### Study population

ART-treated PLHIV were recruited from the hospital located in Pernambuco, Northeast Brazil: the Instituto de Medicina Integral Professor Fernando Figueira (IMIP). Eligible participants were adults (≥ 18 years), treated with ART for at least 12 months with consistent adherence and prolonged undetectable viral loads. Conversely, individuals with cancer, autoimmune diseases, pregnancy, or a history of intravenous drug use during the ART were excluded. All participants provided written informed consent prior to enrollment, including consent for the collection of blood samples and the use of their data for research purposes. The study complied with ethical standards and received approval from IMIP Ethics Committee (protocol code: 65468222.3.1001.8807).

### DNA extraction and quantification

Genomic DNA was extracted from 500 µL of peripheral blood using a modified *in-house* mini-salting-out protocol [25]. DNA quantification was performed using a NanoDrop 2000 spectrophotometer (ThermoFisher), and its integrity was assessed by 1.5% agarose gel electrophoresis.

### ***CCR5***Δ32 genotyping

*CCR5*Δ32 genotyping was performed by polymerase chain reaction (PCR) using the following primers: 5’-GTCTTCATTACACCTGCAGCTCT-3’ (forward primer) and 5’-CACAGCCCTGTGCCTCTT-3’ (reverse primer). PCR was performed in a 25 µL reaction containing 100 ng of genomic DNA, 2.5 µL of 10x buffer, 0.8 µL of dNTPs (2 mM), 0.4 µL of each primer (10 µM), 1 µL of MgCl_2_ (25 mM), 0.2 µL of Taq polymerase (5 U/µL; Affymetrix USB), and ultrapure water to volume. The reaction cycles included an initial denaturation at 95 °C for 10 min, followed by 38 cycles of 95 °C for 30 s, 65 °C for 30 s, and 72 °C for 60 s, with a final extension at 72 °C for 7 min. Amplification products were loaded in 3% agarose gel stained with ethidium bromide and visualized under ultraviolet light. Genotypes were determined based on band patterns: a single 185 bp band indicated homozygosity for the wild-type allele (wt/wt), the presence of both 185 bp and 153 bp bands indicated heterozygosity (wt/Δ32), and a single 153 bp band corresponded to homozygosity for the *CCR5*Δ32 mutation (Δ32/Δ32).

### Peripheral blood mononuclear cells isolation

Peripheral blood mononuclear cells (PBMC) were isolated by Ficoll-Paque Plus density gradient centrifugation and washed twice with PBS (1x). The PBMCs were then resuspended in FACS buffer (3% BSA, 0.01% sodium azide, and PBS 1x). Cell viability was estimated using the Trypan blue (0.4%) exclusion method.

### CD4+ T cell phenotyping

PBMCs were stained with a combination of fluorescent monoclonal antibodies for 20 min at room temperature and protected from light. After staining, cells were washed with FACS buffer, fixed with 1% PBS-formaldehyde, and acquired using a Accuri C6 cytometer (BD Biosciences). For each sample, 100.000 events were recorded and gated to identify specific CD4+ T cell subsets: recent thymic emigrant (RTE) CD4+ T cells (CD4+/ CD45RA+ CD31+), naive CD4+ T cells (CD4+/ CD45RA+ CD62L+), central memory CD4+ T cells (CD4+/ CD45RA- CD62L+), effector memory CD4+ T cells (CD4+/ CD45RA- CD62L-), and effector CD4+ T cells (CD4+/ CD45RA+ CD62L-). The fluorescent monoclonal antibodies used included APC-CD4 (EDU-2), BB515-CD4 (RPA T4), PE-CD31 (WM59), PerCP-Cy5.5-CD45RA (HI100), and APC-CD62L (DREG-56), all sourced from BD Biosciences and BioAlbra. Data was analyzed using FlowJo software, version 10.

### Statistical analysis

Chi-squared test was used to evaluate categorical variables. Moreover, the Shapiro-Wilk test was utilized to evaluate whether numerical variables followed a Gaussian distribution. Variables conforming to a Gaussian distribution were compared across groups using the Student’s T-test and exhibited as mean ± standard deviation (SD). For non-Gaussian variables, the Mann-Whitney test was used, with results demonstrated as median and interquartile range (IQR). The statistical significance level (α) was set at 0.05 for all tests. Statistical analyses were performed using GraphPad Prism, version 8.0.1.

## Results

**Fig. 1:**
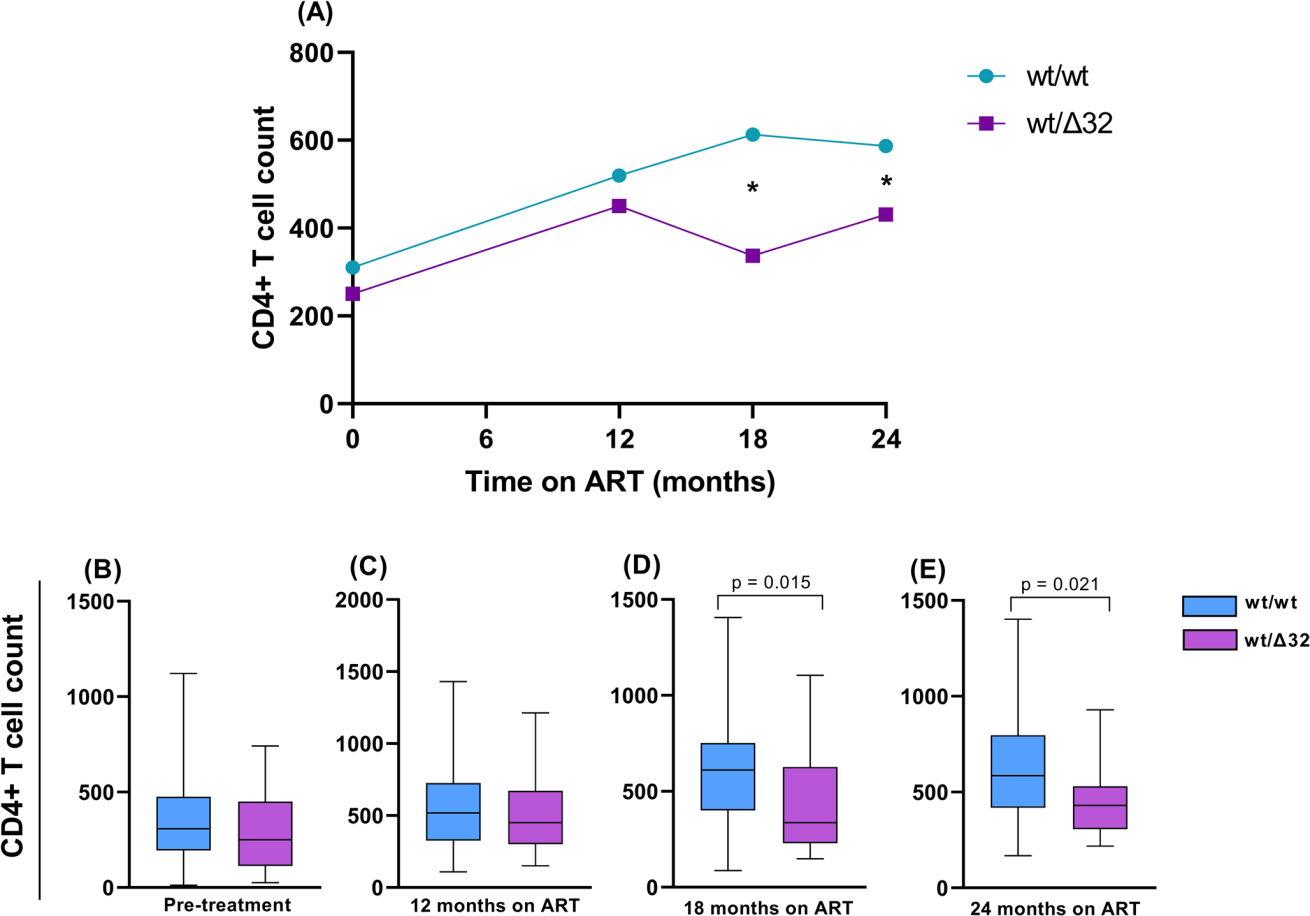
Median of absolute CD4+ T cell counts in PLHIV with wt/wt and wt/Δ32 genotypes at pre-ART, 12, 18, and 24 months of ART. ART: antiretroviral therapy; wt: wild type; Δ32: delta 32 deletion; *: *p* < 0.05. Data are presented as median given the non-normal distribution of CD4+ T cell counts

The study population consisted of 236 ART-treated PLHIV, including 217 with the wt/wt genotype and 19 with the wt/Δ32 genotype. We analyzed the clinical variables, including pre-treatment CD4+ T cell count, CD4+ T cell levels after 12, 18 and 24 months of ART, as well as the ART detailed regimens. Although both groups exhibited CD4+ T cell recovery following ART initiation, individuals with the wild-type homozygous genotype (wt/wt) consistently showed higher absolute CD4+ T cell counts compared to *CCR5*Δ32 heterozygotes (wt/Δ32) throughout the study period (Fig. 1A). At pre-treatment and after 12 months of ART, this difference was not statistically significant (Fig. 1B-C). However, at both 18 and 24 months of ART, the wt/wt group exhibited significantly higher CD4+ T cell counts compared to the wt/Δ32 group (Fig. 1D-E). Regarding other variables, no statistically significant differences were observed between the wt/wt and wt/Δ32 groups (Table 1).

**Table 1:** Clinical characteristics of ART-treated PLHIV based on their genotype (wt/wt vs. wt/Δ32)

Variables		wt/wt *n* = 217 (%)	wt/Δ32 *n* = 19 (%)	*p*-value
Sex^a^	Male Female	106 (48.85) 111 (51.15)	10 (52.63) 9 (47.37)	0.8138
Pre-treatment CD4 + T cell count (cells/µL)*		310.0 (195.0–478.0)	250.5 (112.8–450.3)	0.436
Pre-treatment CD4 + T cell count (%)*		17.15 (13.99–21.81)	15.56 (11.74–21.09)	0.476
CD4 + T cell count after 12 months of ART (cells/µL)*		519.4 (325.5–730.5)	450.0 (300.0–673.0)	0.566
CD4 + T cell count after 12 months of ART (%)*		28.81 (25.10–32.84)	27.51 (24.61–31.83)	0.588
CD4 + T cell count after 18 months of ART (cells/µL)*		613.3 (402.5–766.3)	337.0 (228.8–627.3)	**0.015**
CD4 + T cell count after 18 months of ART (%)*		30.06 (25.42–33.21)	23.99 (21.59–30.45)	**0.018**
CD4 + T cell count after 24 months of ART (cells/µL)*		587.0 (418.0–799.0)	431.0 (307.0–531.0)	**0.021**
CD4 + T cell count after 24 months of ART (%)*		31.79 (28.58–35.81)	28.83 (26.47–30.74)	**0.0225**
Detailed ART regimens, NRTI + 3TC + (third option)^b^	ABC+ 3TC + DTG	1 (0.5)	0 (0.0)	0.314
	TDF + 3TC + ATV/r	12 (7.0)	0 (0.0)	
	TDF + 3TC + LPV/r	2 (1.1)	0 (0.0)	
	TDF + 3TC + DTG	12 (6.9)	1 (6.3)	
	TDF + 3TC + EFZ	17 (10.0)	4 (25.0)	
	AZT + 3TC + LPV/r	48 (27.9)	2 (12.5)	
	AZT + 3TC + NVP	6 (3.4)	2 (12.5)	
	AZT + 3TC + EFZ	65 (38.0)	5 (31.2)	
	AZT + 3TC + ATV/r	8 (4.6)	2 (12.5)	
	AZT + 3TC + FPV/r	1 (0.6)	0 (0.0)	
ART regimens, stratified by classes^b^	2 NRTI + INI	12 (6.8)	1 (6.3)	0.401
	2 NRTI + IP/r	75 (41.6)	4 (25.0)	
	2 NRTI + NNRTI	93 (51.6)	11 (68.7)	

* Wilcoxon-Mann-Whitney test (Shapiro-Wilk: <0.05), values displayed as median (IQR)

^a^ Fisher exact test

^b^ Chi-squared test

3TC: lamivudine; ABC: abacavir; ART: antiretroviral therapy; ATV/r: ritonavir-boosted atazanavir; AZT: zidovudine; DTG: dolutegravir; EFZ: efavirenz; INI: integrase inhibitor; IQR: interquartile range; NNRTI: non–nucleoside reverse transcriptase inhibitor; NRTI: nucleoside reverse transcriptase inhibitor; PI/r: ritonavir-boosted protease inhibitor

**Fig. 2:**
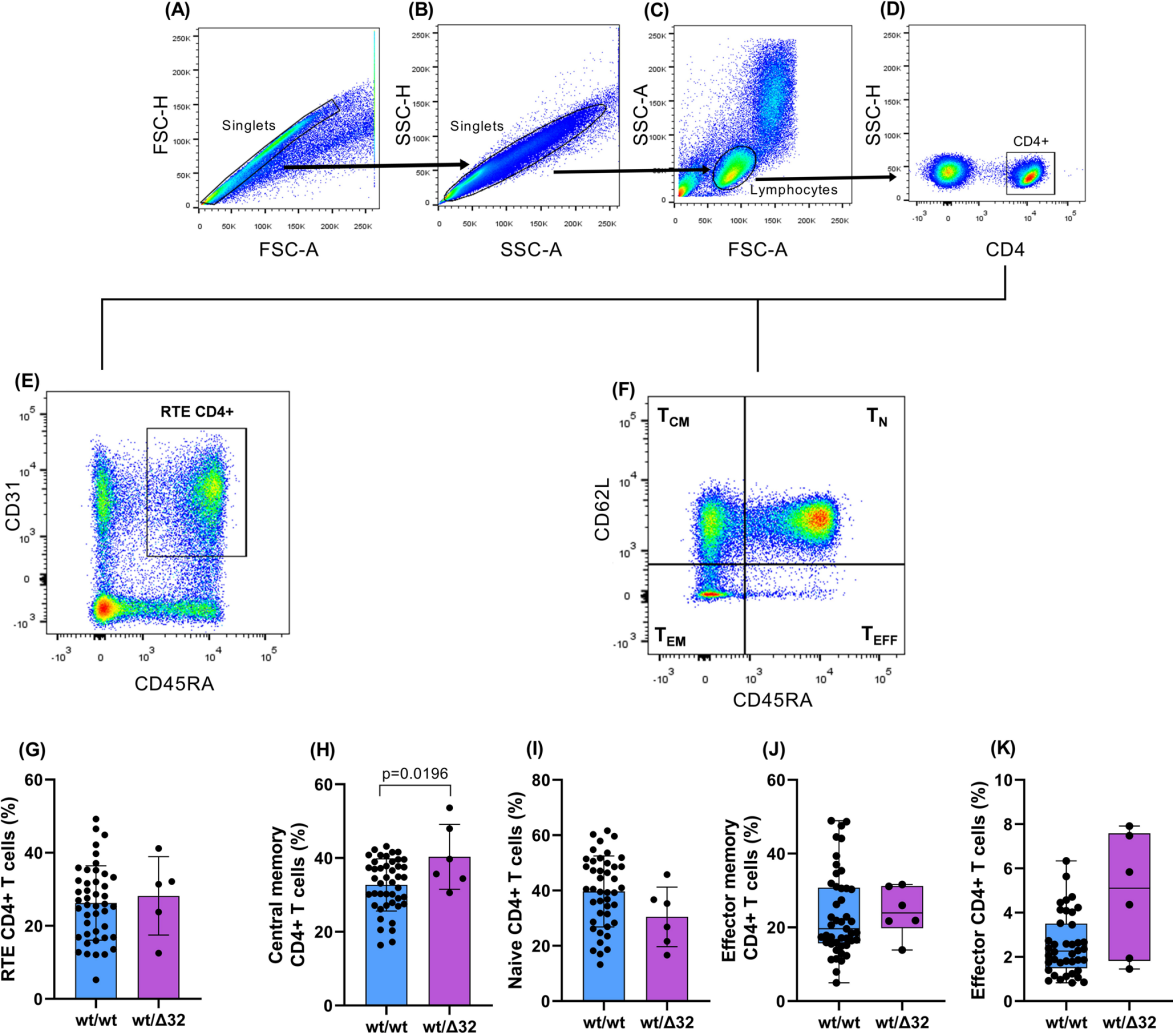
Evaluation of the frequencies of CD4+ T cell subsets among PLHIV after 24 months on ART according to *CCR5*Δ32 genotype (wt/wt vs. wt/Δ32). Displayed in the representative gating strategy (A–F) and CD4+ T cell subset frequencies (G–K). Heterozygous individuals (wt/Δ32) demonstrated a statistically higher frequency of central memory CD4+ T cells compared to wild-type homozygotes (40.33 ± 8.79 vs. 32.71 ± 7.06; *p* = 0.0196; H) and a statically trend toward increased effector CD4 + T cells (5.100 [1.818–7.588] vs. 2.260 [1.485–3.503]; *p* = 0.0459; K). No statistically significant differences were observed between genotypes, heterozygotes vs. wild-type homozygotes, respectively, for RTE CD4+ T cells (28.19 ± 10.72 vs. 26.24 ± 10.19; *p* = 0.6876; G), naive CD4 + T cells (30.47 ± 10.76 vs. 39.58 ± 13.13; *p* = 0.1127; I), or effector memory CD4+ T cells (23.93 [19.77–31.25] vs. 19.61 [15.70–30.80]; *p* = 0.4835; J). Data are presented as mean ± standard deviation for normally distributed variables (G–I), and as median with interquartile range for non-normally distributed variables (J–K) wt: wild type; Δ32: delta 32 deletion. RTE: recent thymic emigrants; T_N_: naive CD4+ T cell: T_CM_: central memory CD4+ Tcell; T_EM_: effector memory CD4+ Tcell; T_EFF_: effector CD4+ T cell

We evaluated the distribution of CD4+ T cell subsets among PLHIV after 24 months on ART according to *CCR5*Δ32 genotype (wt/wt vs. wt/Δ32), as displayed in the representative gating strategy (Figs. 2A–F) and CD4+ T cell subset frequencies (Figs. 2G–K). Heterozygous individuals (wt/Δ32) demonstrated a statistically higher frequency of central memory CD4+ T cells compared to wild-type homozygotes (40.33 ± 8.79 vs. 32.71 ± 7.06; *p* = 0.0196; Fig. 2H) and a statically trend toward increased effector CD4+ T cells (5.100 [1.818–7.588] vs. 2.260 [1.485–3.503]; *p* = 0.0459; Fig. 2K). No statistically significant differences were observed between genotypes, heterozygotes vs. wild-type homozygotes, respectively, for RTE CD4+ T cells (28.19 ± 10.72% vs. 26.24 ± 10.19%; *p* = 0.6876; Fig. 2G), naive CD4+ T cells (30.47 ± 10.76% vs. 39.58 ± 13.13%; *p* = 0.1127; Fig. 2I), or effector memory CD4+ T cells (23.93 [19.77–31.25] vs. 19.61 [15.70–30.80]; *p* = 0.4835; Fig. 2J).

## Discussion

Central memory CD4+ T cells, which express high levels of CCR5, are essential for preserving immune homeostasis due to their high proliferative capacity and central role in coordinating adaptive immune responses [14, 26]. These cells are also a major target for HIV infection [27]. Notably, Yang et al. demonstrated that high CCR5 expression on central memory CD4+ T cells is associated with worse prognosis and faster disease progression [28]. We suggest that PLHIV carrying the *CCR5*Δ32 polymorphism experience lower viral infection rates in these cells, maintaining a larger central memory CD4+ T cell pool during infection. Supporting this hypothesis, our data demonstrated that heterozygous individuals exhibited a significantly higher frequency of central memory CD4+ T cells after 24 months of ART compared to wild-type homozygotes. Additionally, heterozygotes exhibited a higher frequency of effector CD+ T cells, likely due to reduced HIV-mediated apoptosis or altered T cell differentiation influenced by lower CCR5 signaling [16]. These results underscore the protective effect of the *CCR5*Δ32 allele on specific T cell subsets essential for immune response, function and homeostasis.

Interestingly, while *CCR5*Δ32 heterozygotes demonstrated a statistically higher frequency of central memory CD4+ T cells, these individuals exhibited statistically lower absolute CD4+ T cell counts after 18 and 24 months of ART. Although this may appear contradictory, it is essential to distinguish between the frequency of specific CD4+ subsets and the overall absolute count of CD4+ T cells. One possible explanation is that reduced CCR5 expression is protective against HIV initial cellular entry and destruction, but also may impair the trafficking, proliferation, or homeostatic expansion of CD4+ T cells during immune reconstitution [29]. CCR5 plays an important role in the response to CCR5-binding chemokines such as CCL3, CCL4, and CCL5, which are involved in T cell migration and IL-2-mediated proliferation [30, 31]. In this context, the reduced CCR5 expression in heterozygotes could limit the chemokine-driven expansion of CD4+ T cells following viral suppression, resulting in lower CD4+ total counts. This hypothesis is supported by previous studies that associated *CCR5*Δ32 heterozygosity with immunological nonresponse in PLHIV on ART [32]. Immunological non-responders (INR) are PLHIV on ART who achieve virological suppression but do not adequately restore CD4+ T cell counts, making immunological nonresponse a persistent challenge in HIV treatment [33, 34]. Guedes et al. reported that INR individuals exhibit a higher frequency of central memory CD4+ T cells compared to immunological responders [35]. This could be attributed to higher levels of viral replication in HIV reservoirs and increased immune activation in wild-type homozygotes, two key factors influencing immune response in PLHIV [36, 37].

Our findings suggest that the *CCR5*Δ32 polymorphism could mitigate the depletion of central memory CD4+ T cells, thereby improving immune response outcomes in PLHIV on ART. However, the reduced overall CD4+ T cell recovery in heterozygotes may reflect a dual effect of the polymorphism: protection against viral entry and cellular depletion, but potential limitation in immune reconstitution that could explain conflicting evidence regarding its association with immunological response in PLHIV on ART. Some studies have associated *CCR5*Δ32 heterozygosity with a higher risk of immunological nonresponse [32, 38], while others have shown improved subset preservation and immune response [39, 40]. Our findings bridge these perspectives, suggesting that although central memory CD4+ T cells are better preserved in heterozygotes, this does not necessarily translate into a more robust quantitative immune reconstitution. This raises interesting questions about the balance between preserving specific subsets of CD4+ T cells and the overall reconstitution of CD4+ T cell counts: does the preferential preservation of central memory CD4+ T cells serve as a compensatory mechanism that fails to effectively support overall CD4+ T cell recovery? Further studies are needed to investigate the role of different chemokine pathways or thymic output in influencing these dynamics.

No statistically significant differences were observed in the frequencies of RTE, naive, or effector memory CD4+ T cell subsets in *CCR5*Δ32 heterozygous PLHIV after 24 months of ART. This probably reflects the higher expression of CCR5 on central memory CD4+ T cells, making them more susceptible to the protective effect of *CCR5*Δ32 allele against HIV-mediated depletion [41]. However, since these subsets have also associated with immune response in PLHIV on ART [42, 43], further studies involving larger cohorts and more advanced immunophenotyping are warranted to clarify these dynamics.

This study has some limitations, most importantly the relatively small number of heterozygous individuals, which inherently constrains the interpretation of the immunophenotyping data. In fact, this low frequency of heterozygous individuals is consistent with the reduced frequency of the deletion in PLHIV, which is likely due to its protective effect against HIV infection [44, 45]. However, the limited sample size, particularly in the flow cytometry analyses, compromises the robustness of statistical inferences and warrants cautious interpretation of parameters exhibiting considerable biological variability. Our analyses suggest an association between *CCR5*Δ32 heterozygosity and high frequency of central memory CD4+ T cells after 24 months of ART treatment. These observations should be considered as hypothesis-generating rather than definitive, due to the small number of participants in the wt/Δ32 group (5–6 individuals for the immunophenotyping data). Nevertheless, given the limited number of studies addressing the impact of *CCR5*Δ32 on CD4+ T cell dynamics in ART-treated PLHIV, our results contribute preliminary insights into the potential role of host genetic factors in modulating immune responses to ART.

## Data Availability

No datasets were generated or analysed during the current study.
